# Lower Hospitalization Rates During Fluid Management with Relative Blood Volume Monitoring: A Retrospective Cohort Analysis

**DOI:** 10.3390/jcm15155821

**Published:** 2026-07-25

**Authors:** Linda H. Ficociello, Meijiao Zhou, Mianli Xiao, Hao Han, Michael S. Anger, Jeffrey L. Hymes, Dinesh Chatoth

**Affiliations:** 1 Renal Research Institute, 920 Winter St, Waltham, MA 02451, USA; 2 College of Public Health, University of South Florida, Tampa, FL 33612, USA; 3 Fresenius Medical Care, Waltham, MA 02451, USA

**Keywords:** hemodialysis, hospitalization, observational, relative blood volume monitoring, ultrafiltration

## Abstract

**Background:** Effective fluid management is critical for improving outcomes in patients undergoing chronic hemodialysis (HD). Relative blood volume monitoring (RBVM) supports the optimization of ultrafiltration rate adjustments; however, real-world evidence evaluating its impact on hospitalizations in chronic HD is limited. **Methods:** We conducted a retrospective, propensity score–matched observational cohort study comparing patient outcomes across 165 Fresenius Kidney Care clinics with high RBVM utilization (HI-RBVM; >90% of treatments) matched to 165 clinics not using RBVM (NO-RBVM). Data were collected from July to December 2022. Primary outcomes were patient-level all-cause and fluid-related hospitalizations and hospitalization days over a 6-month follow-up. Multivariable scaled Poisson regression models were used to estimate adjusted incidence rates, rate ratios, and rate differences. Secondary outcomes included patient-level changes in monthly clinical measurements. **Results:** 24,958 patients were included (11,840 across HI-RBVM clinics; 13,118 across NO-RBVM clinics). Adjusted incidence rates were lower in the HI-RBVM group compared to the NO-RBVM group for all-cause hospitalizations (1.57 vs. 1.70 per person-year [ppy]), fluid-related hospitalizations (0.30 vs. 0.35 ppy), and hospitalization days (9.60 vs. 10.8 ppy), corresponding to 119 fewer hospital days per 100 patient-years (95% CI: –184, −54; *p* < 0.001). Among patients with dialysis vintage ≤6 months, those treated in HI-RBVM clinics had smaller increases in pre-HD weight (–0.03 kg vs. +0.46 kg; *p* = 0.008) and ultrafiltration volume (0.11 L vs. 0.21 L; *p* = 0.004), and underwent more frequent estimated dry weight (EDW) adjustments (mean 7.73 vs. 6.61; *p* < 0.001). **Conclusions:** Among HD patients, high RBVM utilization at the clinic level was associated with lower rates of hospitalization. Prospective or quasi-experimental studies are needed to determine causality.

## 1. Introduction

In the United States, the prevalence of chronic kidney disease (CKD) was estimated to be 14% in 2020, and from 2002 to 2022, the number of individuals with newly registered end-stage renal disease (ESRD) increased from 99,956 to 131,194 [1]. Growing evidence demonstrates that excessive interdialytic weight gain, fluid overload, intradialytic hypotension, and suboptimal ultrafiltration rates can all negatively impact clinical outcomes in patients on chronic hemodialysis (HD) [2,3,4,5,6]. To improve clinical outcomes, the use of hemodialysis monitoring technologies to assist with fluid management and the determination of euvolemia have been suggested [7]. Inadequate removal of fluid and resultant hypervolemia is a risk factor for heart failure, the most significant cause of mortality in HD patients [8]. Both the amount of fluid removed and the rate of removal (i.e., ultrafiltration rate) may impact treatment outcomes. High ultrafiltration rates are associated with increased all-cause and cardiovascular-related mortality, possibly resulting from transient cardiac ischemia or myocardial stunning [4,9,10,11]. Prior research has shown a positive association between enhanced fluid management and improved outcomes, including reductions in hospitalization [12,13,14,15].

Relative blood volume monitoring (RBVM) can provide information to assist the clinician with determining ultrafiltration rate adjustments, which are needed during dialysis treatment to remove an optimal amount of fluid at the most tolerable rate for the patient. In a retrospective analysis of a fluid management quality improvement project involving 17 dialysis facilities that implemented RBVM, specific hourly intradialytic relative blood volume (RBV) ranges were associated with lower all-cause mortality in chronic HD patients, supporting the potential of RBVM as an indicator of adequate volume management [16]. An additional quality improvement initiative conducted across 15 dialysis facilities in North Texas found that facilities implementing RBVM, alongside an education program, demonstrated greater reductions in all-cause and fluid-related hospitalizations over six months compared with facilities only receiving the education program [14]. Several additional studies have demonstrated improvements in fluid management with RBVM, suggesting RBVM could have a positive impact on selected clinical outcomes [15,17,18,19,20]. Reduced intradialytic symptoms such as lightheadedness, muscle cramping, and nausea with the use of RBVM has also been previously reported in a small study [19]. Furthermore, data from a single dialysis facility identified an association between RBVM and reductions in post-dialysis systolic blood pressure (SBP), as well as longer dialysis treatment times, which may reflect better tolerance of treatments [15]. Despite this, the CLIMB randomized controlled trial, which is to date the most rigorous study evaluating the use of RBVM in HD patients found that RBVM did not reduce hospitalizations and was unexpectedly associated with increased mortality, raising questions regarding the clinical utility of RBVM [21]. Real-world data on the use of RBVM and its potential impact on hospital admissions in the chronic HD setting is limited and may provide further insights into associations between RBVM implementation and outcomes [14,15,16,17,18,19,20].

To evaluate the associations between high clinic-level RBVM utilization and hospitalization outcomes, we conducted a retrospective observational analysis to compare hospitalization outcomes over a 6-month follow-up among Fresenius Kidney Care (FKC) clinics with a high utilization of RBVM, and propensity score matched clinics not using RBVM. Since patients with longer dialysis vintage may have already achieved relative stabilization of monthly clinical measurements, we additionally examined a subset of patients with a dialysis vintage ≤6 months. Among these patients, we examined monthly average clinical measurements and the number of estimated dry weight (EDW) changes. This cohort may provide more opportunities to observe differences in volume-management practices associated with RBVM adoption.

## 2. Materials and Methods

### 2.1. Study Design and Data Source

This retrospective analysis evaluated the use of RBVM in FKC clinics and compared patient outcomes from 165 clinics in the United States with high utilization of RBVM (HI-RBVM; >90% of HD treatments using RBVM) with propensity score matching to 165 FKC clinics that did not use RBVM (NO-RBVM). Propensity scores were calculated using a generalized linear model that included clinic-level patient characteristics (patient census, average age, percent of male patients, percent of black patients, percent of Medicare patients, percent of diabetes, percent of catheter use, ESRD network affiliation). Clinics were matched using nearest-neighbor matching with exact matching on ESRD network affiliation. RBVM was conducted using Crit-Line^®^ (CLiC™; Crit-Line in a Clip, Fresenius Medical Care, Waltham, MA, USA) devices integrated into 2008T dialysis machines. All data included in the analysis was extracted from the FKC data warehouse, fully de-identified, and collected as part of routine clinical care from 1 July 2022–31 December 2022. The primary outcome of interest was hospitalization rates, while secondary outcomes included other clinical measurements. This study was reviewed by an independent review board (New England Independent Review Board (NEIRB)/WCG IRB, Needham, MA, USA) and was granted an exempt status determination under the common rule and applicable guidance.

### 2.2. Relative Blood Volume Monitoring Device

Crit-Line^®^ devices are Food and Drug Administration 510K cleared devices that continuously and non-invasively measure hematocrit to calculate hemoglobin and percentage change in RBV, along with oxygen saturation during HD treatment. The use of these devices and the procedures followed by nurses and technicians has been previously described [15,22]. Each dialysis session was initiated with a physician-ordered target weight and ultrafiltration goal. In addition to standard assessments of vital signs and clinical volume status, percentage change in RBV and hematocrit was recorded every 30 min. At the discretion of the physician, the ultrafiltration rate was adjusted, up to 200 mL every 30 min, to obtain a gradual decrease in percentage change in blood volume and to maintain a patient-tolerable ultrafiltration rate while maximizing fluid removal [15,22]. Target weights for subsequent sessions could be adjusted using hematocrit-based assessments of vascular compartment refill at the end of HD [15].

### 2.3. Statistical Analysis

A total of 165 HI-RBVM FKC clinics were propensity score matched to 165 NO-RBVM FKC clinics through nearest neighbor matching. Covariate balance was assessed using absolute standardized mean differences. To account for the hospital stay duration during the observation period, follow-up days were adjusted by subtracting the length of hospital stays in the all-cause and fluid-related hospitalizations models. The characteristics of the included subjects were described using counts and percentages (*n*, %) for categorical variables and means with standard deviations (mean, SD) for continuous variables. A generalized linear regression model with a Poisson distribution (log link function) and log-transformed follow-up days as the offset was employed to calculate the unadjusted and adjusted incidence rates, rate ratios (RR), and rate differences for all-cause hospitalization, fluid-related hospitalization, and hospital stays between the RBVM use groups (HI-RBVM vs. NO-RBVM). A scale parameter was used to account for potential overdispersion in the count data. To account for any patient-level residual confounding after our clinic-level match, we controlled for the following covariates in our final outcomes model: age, dialysis vintage, gender, race, Hispanic ethnicity, insurance type, catheter use, congestive heart failure (CHF), and diabetes. We also presented *p* values from the scaled Poisson models adjusting for the clinic clustering effect, by using generalized estimating equation models with robust standard errors, specifying clinic as the clustering unit. As a sensitivity analysis, a negative binomial regression model was performed to evaluate the robustness of the findings.

We also assessed monthly average clinical measurements between HI-RBVM and NO-RBVM groups, including treatment time, pre-HD weight, post-HD weight, pre-HD SBP, post-HD SBP, EDW, interdialytic weight gain, ultrafiltration volume (UFV), and ultrafiltration rate (UFR). The differences in clinical measurements between Month 1 and Month 6 were compared using paired *t*-tests. Repeated measures analyses were conducted to compare the monthly changes within and between RBVM groups. Additionally, the number of EDW adjustments during the observation period was calculated and compared between the RBVM groups using *t*-tests. All analyses were conducted with SAS (SAS Enterprise Guide 7.1; SAS Institute Inc., Cary, NC, USA).

## 3. Results

### 3.1. Study Population and Characteristics

Absolute standardized mean differences in the matched variables ranged from 0.07 to 0.64 before matching, and were ≤0.11 after matching, indicating acceptable balance between HI-RBVM and NO-RBVM groups (Supplementary Table S1). Following propensity score matching at the clinic level, 24,958 ESRD patients were included in the analysis, with 11,840 from HI-RBVM clinics and 13,118 from NO-RBVM clinics (Table 1). Baseline characteristics were generally well-balanced between groups. The mean age was 64.2 (±13.9) years in both groups, and gender distribution was also comparable, with females representing 41.1% and 40.9% of the HI-RBVM and NO-RBVM groups, respectively. Racial distribution showed minor differences, with Black patients comprising 21.9% of the HI-RBVM group and 22.4% of the NO-RBVM group, while White patients accounted for 67.6% and 66.5% of each group, respectively. The proportion of Hispanic patients was slightly lower in the HI-RBVM group (18.4%) compared to the NO-RBVM group (21.0%).

Dialysis vintage was slightly longer in the HI-RBVM group, averaging 3.68 (±4.09) years compared to 3.48 (±3.94) years in the NO-RBVM group. Access type distributions were comparable, though arteriovenous fistula (AVF) use was slightly higher in the HI-RBVM group (56.1% vs. 53.7%). Medicare was the predominant insurance type (76.3% in HI-RBVM vs. 78.6% in NO-RBVM), with similar proportions of Medicaid and commercial coverage. The causes of ESRD were similar between groups, with diabetes being the primary cause in both groups, followed by hypertension. Comorbidities were similar between groups, with comparable proportions of hypertension, diabetes, CHF, and chronic obstructive pulmonary disease (COPD) observed.

### 3.2. Hospitalization Outcomes

The total follow-up period amounted to 4517 person-years in the HI-RBVM group and 4640 person-years in the NO-RBVM group. The total number of all-cause hospitalizations was 6668 for the HI-RBVM group compared to 7416 for the NO-RBVM group. For fluid-related hospitalizations, the HI-RBVM group experienced 1176 events, whereas the NO-RBVM group experienced 1396 events.

The unadjusted incidence rate of all-cause hospitalizations was higher in the NO-RBVM group compared to the HI-RBVM group (1.65 per person-year [ppy; 95% CI: 1.58, 1.71] vs. 1.52 ppy [95% CI: 1.45, 1.58]; *p* = 0.004) (Table 2). *p*-values adjusted for clinic clustering are also presented in Table 2 and were all significant at *p* < 0.05. The unadjusted incidence rate of hospitalization days was lower in the HI-RBVM group compared with the NO-RBVM group (9.43 vs. 10.6 ppy, respectively), corresponding to approximately 118 fewer hospital days per 100 patient-years (95% CI: −184, −53; *p* = 0.004). Likewise, the unadjusted incidence rate for fluid-related hospitalizations was higher in the NO-RBVM group than the HI-RBVM group (0.31 ppy [95% CI: 0.29, 0.34] vs. 0.27 ppy [95% CI: 0.24, 0.29]; *p* = 0.02). Diagnostic codes for fluid-related hospitalization can be found in Supplemental Box S1.

After multivariable adjustment for age, dialysis vintage, gender, race, Hispanic ethnicity, primary insurance, catheter use, comorbid CHF, and diabetes, the findings remained consistent (Table 2). The adjusted incidence rate of all-cause hospitalizations was higher in the NO-RBVM group as compared to the HI-RBVM group (1.70 ppy [95% CI: 1.58, 1.83] vs. 1.57 ppy [95% CI: 1.46, 1.69]; *p* = 0.004). The adjusted incidence rate of hospitalization days was lower in the HI-RBVM group compared with the NO-RBVM group (9.60 vs. 10.8 ppy), corresponding to an adjusted rate difference of 119 fewer hospital days per 100 patient-years (95% CI: −184, −54; *p* < 0.001). Further, the adjusted incidence rate for fluid-related hospitalizations was higher in the NO-RBVM group than the HI-RBVM group (0.35 ppy [95% CI: 0.30, 0.41] vs. 0.30 ppy [95% CI: 0.26, 0.35]; *p* = 0.01). Results from the negative binomial sensitivity analysis (Supplementary Table S2) were consistent with those from the primary scaled Poisson model, although confidence intervals were wider and comparisons in hospital days no longer reached statistical significance. Overall, the direction and magnitude of the estimated rate ratios were similar, indicating that different approaches accounting for overdispersion did not materially affect the findings.

### 3.3. Monthly Average Clinical Measurements and Differences

The monthly average clinical measurements for the HI-RBVM and NO-RBVM groups were recorded over six months, with differences between Month 1 and Month 6 compared across the groups (Table 3). The 1724 subjects included in this analysis were those with a dialysis vintage of less than 6 months, as newly initiated HD patients may better reflect the changes from RBVM.

The treatment time decreased slightly in both groups over the six months, however the difference between groups was not statistically significant between the RBVM groups (*p* = 0.61). For pre-HD weight, the NO-RBVM group showed a small but statistically significant increase of 0.46 kg (*p* = 0.003) while the HI-RBVM group maintained a similar weight with a −0.03 kg decrease (*p* = 0.85), and a statistically significant between-group difference (*p* = 0.008). Post-HD weight differences were 0.25 kg (*p* = 0.11) in the NO-RBVM group and −0.15 kg (*p* = 0.38) in the HI-RBVM group, with no significant difference between groups (*p* = 0.06). Interdialytic weight gain (IDWG) remained relatively stable over time, with no clinically significant differences between the groups (*p* = 0.09). Slight decreases in EDW were observed for both groups, however differences between each group were not statistically significant (*p* = 0.18). A lesser increase in UFV was observed in the HI-RBVM group (0.11 L, *p* < 0.001) compared to the NO-RBVM group (0.21 L, *p* < 0.001), with a significant difference between groups observed (*p* = 0.004). Ultrafiltration rate (UFR) increased over time in the HI-RBVM group (0.38 mL/kg/h, *p* < 0.001) and the NO-RBVM group (0.71 mL/kg/h, *p* < 0.001), the changes were statistically significant (*p* = 0.006) between groups. Both groups demonstrated significant increases in pre-HD SBP and significant decreases in post-HD SBP, however differences between each group were not statistically significant for both pre- and post-HD SBP. Decreases in the percentage of patients on hypertension medication were observed in both groups, the reductions were higher in the HI-RBVM group (*p* = 0.02). Similarly, the percentage of patients on ≥3 hypertension medications significantly decreased in each group; however, the between-group difference was not statistically significant (*p* = 0.21). Apart from pre-HD weigh, UFV, and UFR, all of which showed significantly smaller changes in the HI-RBVM group, other blood pressure and weight-related parameters did not differ significantly between groups. A higher reduction in hypertension medication was observed in the HI-RBVM group.

Clinical measurement changes were most prominent in the dialysis vintage of ≤6 months. When comparing the monthly average clinical measurements between HI-RBVM and NO-RBVM groups among all patients, and among patients followed-up for 6 months regardless of dialysis vintage, there were no significant differences observed between the two groups over 6 months, except for the hypertension medication changes among all patients. (Supplemental Table S3A,B).

### 3.4. Number of EDW Changes over Time

The number of EDW changes during the 6-month follow-up period among patients with a dialysis vintage of ≤6 months (*n* = 1731) was also assessed in the overall cohort and by subgroup based on hypertension status in the first month (Table 4). Overall, the HI-RBVM group reported a higher mean number of EDW changes (7.73 ± 4.83), than the NO-RBVM group (6.61 ± 4.44; *p* < 0.001). For patients without hypertension in the first month, the HI-RBVM group also reported a higher mean number of EDW changes (7.38 ± 4.71), compared to the NO-RBVM group (6.23 ± 3.88; *p* <0.001). Among patients with hypertension in the first month, the HI-RBVM group reported a mean of 8.04 ± 4.91 EDW changes, while the NO-RBVM group reported a mean of 6.89 ± 4.77 (*p* < 0.001). Similar findings were observed among patients followed for 6 months, regardless of dialysis vintage (*n* = 15,196), where the HI-RBVM group experienced more frequent EDW adjustments than the NO-RBVM group, with statistically significant differences observed irrespective of hypertension status in the first month (Supplemental Table S4).

## 4. Discussion

Optimal fluid management is recognized as one of the primary goals of dialysis care [2]. As previously described, RBV monitors supplement the clinical assessment of fluid status and can assist with fluid management during HD [17,23,24,25]. However, clinical outcomes associated with RBVM among HD patients have not been well characterized [14]. In this large, retrospective analysis of about 25,000 HD patients, we found that patients treated at clinics with high utilization of RBVM using Crit-Line^®^ devices experienced significantly lower rates of all-cause hospitalizations, fluid-related hospitalizations, and hospital days compared to patients at propensity score-matched clinics not using RBVM. These findings remained consistent after adjusting for multiple potential confounders. One plausible explanation is that consistent use of Crit-Line^®^ devices may enable improved fluid management and reduced healthcare utilization. While most clinical parameters showed no significant differences between the HI-RBVM and NO-RBVM groups, there were significant, albeit small, differences in pre-HD weight, UFV, UFR, and hypertension medication usage among patients with dialysis vintage ≤6 months. This difference was not observed in the population that included all dialysis vintage patients. It is possible that Crit-Line^®^ monitoring may help better manage fluid removal and prevent early weight gain in newly initiated patients, who are often at higher risk of volume-related complications [26]. Incorporating RBVM early in dialysis care may support more precise fluid management during this transitionary period. Additionally, HI-RBVM clinics demonstrated more frequent EDW adjustments for patients with dialysis vintage ≤6 months. This finding may reflect a more proactive approach to fluid optimization and more precise management of fluid balance during the early dialysis period when volume status is still being stabilized. However, alternative explanations cannot be excluded such as differences in patient complexity, provider practice patterns, documentation practices across clinics.

The results contrast with earlier studies, such as the CLIMB randomized controlled trial, which reported greater hospitalizations and mortality among patients monitored with Crit-Line^®^ devices as compared to conventional monitoring [21]. Importantly, the present observational study does not override randomized evidence. In the CLIMB study, a patient monitoring and intervention algorithm was developed to assist in managing patients monitored by Crit-Line^®^ devices. Notably, its use was only encouraged and not mandated, which may have led to suboptimal or inconsistent interventions. Furthermore, although the CLIMB trial provides randomized evidence for RBVM use, questions have been raised regarding the generalizability of the study’s findings since hospitalization and mortality rates were unusually low in the conventional monitoring group as compared to the general ESRD population. This suggests the possibility of selection bias or other factors, such as the Hawthorne effect, whereby individuals modify their behavior in response to being observed. While the management of patients using Crit-Line^®^ devices in the CLIMB study was not standardized, it is important not to dismiss the results, as improperly trained staff and high turnover are key challenges that can result in non-standardized use of Crit-Line^®^ devices in clinical practice [22]. Our study specifically evaluated clinics with high Crit-Line^®^ device adoption (>90% of treatments), suggesting that RBVM was routinely available and frequently used as part of standard care in a real-world setting. Nonetheless, device adoption does not necessarily reflect how RBVM data were interpreted or acted upon by clinicians. Our findings align with broader literature emphasizing the importance of precise fluid management in HD patients [2,4,6,27]. The importance of optimal fluid management and its relationship with improved clinical outcomes is well recognized [3,28,29,30]. Optimal fluid management requires clinicians to avoid high ultrafiltration rates, which are associated with increased mortality, while simultaneously ensuring adequate fluid removal [3,4,28,31,32]. Frequent assessment of fluid status, including the use of RBVM, aims to assist clinicians in tailoring fluid removal to the needs of patients [2,6]. By providing real-time, non-invasive hematocrit and RBV trends, Crit-Line^®^ devices offer a diagnostic tool to monitor fluid removal during HD treatment. While the device itself does not dictate interventions, clinics that actively incorporate RBVM into decision-making may be better positioned to avoid both chronic fluid overload and excessive ultrafiltration, thereby improving patient outcomes. The extent to which these potential advantages translate into improved clinical outcomes remains to be defined.

This study has several limitations to consider. First, the study design compared clinics with different RBVM adoption patterns rather than directly comparing patients who had documented RBVM exposure data. Second, the retrospective observational design, in which the exposure and outcomes were assessed concurrently, limits the ability to establish causal relationships between RBVM and the observed reductions in hospitalization rates. Although propensity score matching and multivariable adjustments were applied to minimize confounding, residual confounding from unmeasured variables such as patient adherence, nutritional status, organizational structure, staff training, clinical practice pattern, and overall quality of care cannot be excluded. Additionally, we lacked data on the specific clinical actions taken in response to RBVM with Crit-Line^®^ devices, which may vary between clinics, and relied on electronic health record data which could be subject to documentation errors. Finally, the follow-up period of 6 months may not capture long-term trends in clinical outcomes, suggesting the need for future long-term studies. Despite these limitations, the study included a large, unselected, real-world population of about 25,000 HD patients across 330 managed kidney care clinics which may enhance the generalizability of the findings. Additionally, the use of propensity score matching and multivariable adjustment helped to minimize bias and strengthened the validity of the observed outcomes between RBVM and clinical outcomes.

In conclusion, our results suggest that high RBVM utilization at the clinic level is associated with lower rates of hospitalization in HD patients. These findings highlight the need for future prospective, long-term studies to confirm these associations.

## Figures and Tables

**Table 1:** The characteristics of patients included at the beginning of the study.

Characteristics	HI-RBVM	NO-RBVM
Mean (SD)/*n* (%)	*n* = 11,840	*n* = 13,118
Age	64.2 (13.9)	64.2 (13.9)
Dialysis vintage in years	3.68 (4.09)	3.48 (3.94)
Female	4867 (41.1)	5366 (40.9)
Race		
Black	2592 (21.9)	2941 (22.4)
White	8009 (67.6)	8729 (66.5)
Hispanic	2177 (18.4)	2758 (21.0)
Others	645 (5.4)	578 (4.4)
Unknown	594 (5.0)	870 (6.6)
Access Type		
AVF	6647 (56.1)	7039 (53.7)
AVG	1581 (13.4)	1924 (14.7)
CVCATH	3612 (30.5)	4155 (31.7)
Insurance		
Medicare	9036 (76.3)	10,310 (78.6)
Medicaid	904 (7.6)	981 (7.5)
Commercial	1144 (9.7)	1179 (9.0)
Others	754 (6.4)	644 (4.9)
Unknown	2 (0.02)	4 (0.03)
ESRD Cause		
Diabetes	5377 (45.4)	6121 (46.7)
Hypertension	3196 (27.0)	3708 (28.3)
Glomerulonephritis	753 (6.4)	777 (5.9)
Polycystic	305 (2.6)	321 (2.4)
Others	1856 (15.7)	1753 (13.4)
Unknown	353 (3.0)	438 (3.3)
Comorbidities		
CHF	3152 (26.6)	3332 (25.4)
Diabetes	6743 (57.0)	7491 (57.1)
Hypertension	8703 (73.5)	9514 (72.5)
COPD	1367 (11.5)	1521 (11.6)
On anti-hypertensive medication	9110 (76.9)	10,029 (76.5)
On ≥3 types of anti-hypertensive medications	3803 (32.1)	4179 (31.9)

AVF, arteriovenous fistula; AVG, arteriovenous graft; CHF, congestive heart failure; COPD, chronic obstructive pulmonary disease; CVCATH, central venous catheter; ESRD, end-stage renal disease; HI-RBVM, high utilization of relative blood volume monitoring; NO-RBVM, not using relative blood volume monitoring; SD, standard deviation.

**Table 2:** The results of univariable and multivariable generalized linear regression with Poisson distribution.

Outcome	Incidence Rate ppy		Rate Ratio	Rate Difference	*p*-Value ^1^	*p*-Value ^2^
	HI-RBVM	NO-RBVM	HI-RBVM vs. NO-RBVM	HI-RBVM vs. NO-RBVM	HI-RBVM vs. NO-RBVM	HI-RBVM vs. NO-RBVM
Univariable models						
All-cause hospitalizations	1.52 (1.45, 1.58)	1.65 (1.58, 1.71)	0.92 (0.87, 0.97)	−0.13 (−0.22, −0.04)	0.004	0.04
Hospital days	9.43 (8.99, 9.90)	10.6 (10.2, 11.1)	0.89 (0.83, 0.95)	−1.18 (−1.84, −0.53)	<0.001	0.04
Fluid-related hospitalizations	0.27 (0.24, 0.29)	0.31 (0.29, 0.34)	0.86 (0.76, 0.97)	−0.04 (−0.08, −0.01)	0.02	0.04
Multivariable models						
All-cause hospitalizations	1.57 (1.46, 1.69)	1.70 (1.58, 1.83)	0.92 (0.87, 0.97)	−0.13 (−0.22, −0.04)	0.004	0.03
Hospital days	9.60 (8.81, 10.5)	10.8 (9.90, 11.7)	0.89 (0.84, 0.95)	−1.19 (−1.84, −0.54)	<0.001	0.008
Fluid-related hospitalizations	0.30 (0.26, 0.35)	0.35 (0.30, 0.41)	0.86 (0.77, 0.97)	−0.05 (−0.09, −0.01)	0.01	0.04

^1^ *p* values were from the scaled Poisson model. ^2^ *p* values were from the scaled Poisson model, adjusting for the clinic clustering effect. Univariable: no covariate adjustment; Multivariable: adjusted for age, vintage, gender, race, Hispanic ethnicity, primary insurance, catheter use, comorbid congestive heart failure and diabetes. HI-RBVM, high utilization of relative blood volume monitoring; NO-RBVM, not using relative blood volume monitoring; ppy, per person year.

**Table 3:** Comparison of the monthly average clinical measurements between the HI-RBVM and NO-RBVM groups among patients with a dialysis vintage of ≤6 months and followed-up for 6 months.

Variables	Group	Month 1	Month 2	Month 3	Month 4	Month 5	Month 6	Month 6-1, *p* Value ^1^	*p*-Value ^2^	*p*-Value ^3^
Number of patients	HI-RBVM	836	833	830	830	834	839	836		
	NO-RBVM	888	883	881	885	886	900	888		
Treatment time (min, SD)	HI-RBVM	226.4 (21.9)	226.5 (22.4)	226.2 (22.9)	226 (23.1)	225.7 (22.9)	225.4 (22.9)	−1.03, *p* = 0.02	<0.001	0.61
	NO-RBVM	226.3 (23.5)	226.0 (23.9)	225.6 (23.9)	225.3 (24.1)	224.9 (24.2)	224.8 (24.2)	−1.46, *p* < 0.001	<0.001	
Pre-HD weight (kg, SD)	HI-RBVM	86.3 (23.3)	86.1 (23.1)	86.1 (23.0)	86.3 (23)	86.3 (23)	86.2 (23.1)	−0.03, *p* = 0.85	0.15	0.008
	NO-RBVM	85.8 (24.3)	85.7 (24.2)	85.8 (24.2)	85.8 (24.2)	86.0 (24.1)	86.1 (24.1)	0.46, *p* = 0.003	<0.001	
Post-HD weight (kg, SD)	HI-RBVM	84.2 (22.9)	83.9 (22.7)	83.9 (22.6)	84.1 (22.6)	84.1 (22.5)	84.1 (22.6)	−0.15, *p* = 0.38	0.008	0.006
	NO-RBVM	83.8 (23.8)	83.6 (23.7)	83.7 (23.7)	83.7 (23.7)	83.8 (23.6)	84.0 (23.5)	0.25, *p* = 0.11	<0.001	
IDWG (kg, SD)	HI-RBVM	2.02 (1.00)	2.16 (0.99)	2.19 (0.96)	2.21 (1.03)	2.24 (1.05)	2.15 (1.06)	0.13, *p* < 0.001	<0.001	0.09
	NO-RBVM	1.94 (1.00)	2.09 (1.00)	2.08 (1.07)	2.14 (0.95)	2.24 (1.05)	2.13 (1.04)	0.20, *p* < 0.001	<0.001	
EDW (kg, SD)	HI-RBVM	83.9 (22.8)	83.5 (22.6)	83.4 (22.5)	83.4 (22.4)	83.5 (22.3)	83.4 (22.3)	−0.54, *p* = 0.001	<0.001	0.18
	NO-RBVM	83.6 (23.8)	83.2 (23.6)	83.1 (23.5)	83.1 (23.5)	83.1 (23.5)	83.3 (23.4)	−0.22, *p* = 0.17	<0.001	
Ultrafiltration Volume (L, SD)	HI-RBVM	2.05 (0.94)	2.16 (0.92)	2.2 (0.93)	2.22 (0.99)	2.21 (0.97)	2.16 (0.99)	0.11, *p* < 0.001	<0.001	0.004
	NO-RBVM	1.95 (0.92)	2.08 (0.93)	2.1 (0.95)	2.15 (0.92)	2.19 (0.96)	2.17 (0.98)	0.21, *p* < 0.001	<0.001	
Ultrafiltration Rate (mL/kg/h, SD)	HI-RBVM	6.61 (3.00)	7.01 (2.89)	7.13 (2.96)	7.18 (3.10)	7.14 (3.06)	6.99 (3.20)	0.38, *p* < 0.001	<0.001	0.006
	NO-RBVM	6.36 (2.87)	6.80 (2.85)	6.88 (3.06)	7.09 (2.98)	7.19 (2.95)	7.08 (2.93)	0.71, *p* < 0.001	<0.001	
Pre-HD SBP (mmHg, SD)	HI-RBVM	143.1 (19.3)	144 (19.3)	144.8 (19.8)	145.4 (20.2)	146.1 (20.1)	145.5 (20.4)	2.40, *p* < 0.001	<0.001	0.44
	NO-RBVM	143.7 (18.1)	144.8 (19.2)	145.5 (19)	146.6 (19.2)	147.2 (19.5)	147.3 (19.6)	3.56, *p* < 0.001	<0.001	
Post-HD SBP (mmHg, SD)	HI-RBVM	139.4 (18.0)	138.7 (18.1)	137.8 (17.9)	137 (18)	136.7 (17.8)	136.4 (17.9)	−2.95, *p* < 0.001	<0.001	0.54
	NO-RBVM	139.4 (16.4)	138.4 (16.4)	137.7 (16.6)	137.5 (16.6)	137.2 (17.0)	137.1 (17.5)	−2.33, *p* < 0.001	<0.001	
On HTN medication, %	HI-RBVM	82.8	82.2	81.2	78.8	77.7	77.6	−5.27, *p* < 0.001	<0.001	0.02
	NO-RBVM	83.7	82.7	82.1	82.0	82.1	80.7	−3.04, *p* = 0.003	0.03	
On ≥3 types of HTN medications, %	HI-RBVM	38.2	36.7	33.3	31.7	31.1	29.3	−8.97, *p* < 0.001	<0.001	0.21
	NO-RBVM	36.9	35.4	33.9	33.4	32.7	32.1	−4.85, *p* < 0.001	0.005	

^1^ *p* values were calculated using paired *t*-test to assess the significance of the differences between month 6 and month 1; ^2^ *p* values were calculated using the repeated-measures models to test the significance of monthly changes in each group; ^3^ *p* values were calculated from the repeated-measures models with an interaction term to test whether the monthly changes were significantly different between the HI-RBVM and NO-RBVM groups. CHF, congestive heart failure; EDW, estimated dry weight; HD, hemodialysis; HI-RBVM, high utilization of relative blood volume monitoring; HTN, hypertension; IDWG, interdialytic weight gain; NO-RBVM, not using relative blood volume monitoring; SBP, systolic blood pressure; UFV, ultrafiltration volume.

**Table 4:** Number of EDW changes over time in HI-RBVM and NO-RBVM groups among patients with a dialysis vintage of ≤6 months and followed-up for 6 months.

Study Cohort	Group	Number of EDW Changes, Mean (SD)	*p*-Value
Overall (*n* = 1731)	HI-RBVM	7.73 (4.83)	<0.001
	NO-RBVM	6.61 (4.44)	
By hypertension in the first month			
No hypertension (*n* = 747)	HI-RBVM	7.38 (4.71)	<0.001
	NO-RBVM	6.23 (3.88)	
Hypertension (*n* = 977)	HI-RBVM	8.04 (4.91)	<0.001
	NO-RBVM	6.89 (4.77)	

EDW, estimated dry weight; HI-RBVM, high utilization of relative blood volume monitoring; NO-RBVM, not using relative blood volume monitoring; SD, standard deviation.

## Data Availability

The data underlying the findings described in this manuscript are proprietary and not publicly available. Further inquiries can be directed to Dr. Linda Ficociello at linda.ficociello@rriny.com.

## Supplementary Materials

The following supporting information can be downloaded at https://www.mdpi.com/article/10.3390/jcm15155821/s1: Box S1: Diagnostic codes to define fluid-related hospitalization; Table S1. Absolute standardized mean differences (ASMD) of the matched variables before and after matching; Table S2. The results of univariable and multivariable negative binomial model; Table S3A. Comparison of monthly average clinical measurements between HI-RBVM and NO-RBVM groups among all patients; Table S3B. Comparison of monthly average clinical measurements between HI-RBVM and NO-RBVM groups among patients followed-up for 6 months; Table S4. Number of EDW changes over time in HI-RBVM and NO-RBVM groups among patients with followed-up for 6 months.

## Abbreviations

The following abbreviations are used in this manuscript:

AVF	arteriovenous fistula
CHF	congestive heart failure
CKD	chronic kidney disease
COPD	chronic obstructive pulmonary disease
CVCATH	central venous catheter
EDW	estimated dry weight
ESRD	end-stage renal disease
FKC	Fresenius Kidney Care
HD	hemodialysis
ppy	per-person year
RBV	relative blood volume
RBVM	relative blood volume monitoring
RR	rate ratio
SBP	systolic blood pressure
UFV	ultrafiltration volume
UFR	ultrafiltration rate
