# Supplementary material for: Lower Hospitalization Rates During Fluid Management with Relative Blood Volume Monitoring: A Retrospective Cohort Analysis
Source: J Clin Med. 2026 Jul 25;15(15):5821. doi: 10.3390/jcm15155821 (PMC13466751; doi:10.3390/jcm15155821)
Supplement: Supplementary file 1 [file jcm-15-05821-s001.zip › jcm-4375478-supplementary.pdf]

Supplemental Tables - Legend

I. Supplemental Box S1. Diagnostic codes to define fluid-related hospitalization
II. Supplemental Table S1. Absolute standardized mean differences (ASMD) of the matched variables before and after matching
III. Supplemental Table S2. The results of univariable and multivariable negative binomial model
IV. Supplemental Table S3A. Comparison of monthly average clinical measurements between HI-RBVM and NO-RBVM groups among all patients
V. Supplemental Table S3B. Comparison of monthly average clinical measurements between HI-RBVM and NO-RBVM groups among patients followed-up for 6 months
VI. Supplemental Table S4. Number of EDW changes over time in HI-RBVM and NO-RBVM groups among patients with followed-up for 6 months.

Supplemental Box S1. Diagnostic codes to define fluid-related hospitalization. Both primary and non-primary diagnosis codes were used for hospitalization causes.

"I50.20","I50.21","I50.22","I50.23","I50.30","I50.31","I50.32","I50.33","I50.40", "I50.41", "I50.42", "I50.43", "I50.81", "I50.82", "I50.84", "I50.89", "I50.9", "I11.0","I13.0","I13.10","I13.11","I13.2","I11.0","I13.0","I13.2","I42.0","I50", "E87.7","E87.70","E87.79", "R06.02","R06.00","R60.1","R60.0","R60.9","J81.0","J81.1","I50.1", "J96.00","J96.90","J96","J96.0","J96.00","J96.01","J96.02","J96.1","J96.10","J96.11","J96.12","J96.2","J96.20","J96.21","J96.22", "J96.9","J96.90","J96.91","J96.92"

Supplemental Table S1. Absolute standardized mean differences (ASMD) of the matched variables before and after matching at the clinic-level

Variables used in the propensity score matching	ASMD Before matching	ASMD After matching
Patient census	0.41	0.06
Average age	0.19	0.002
Percent of male	0.15	0.07
Percent of Black	0.64	0.02
Percent of Medicare	0.30	0.10
Percent of diabetes	0.36	0.05
Percent of catheter use	0.42	0.11
ESRD network affiliation	0.07	0

Supplemental Table S2. The results of univariable and multivariable negative binomial models

Outcome	Rate ratio HI-RBVM vs. NO-RBVM	P-value[1] HI-RBVM vs. NO-RBVM	P-value[2] HI-RBVM vs. NO-RBVM
Univariable models			
All-cause hospitalizations	0.92 (0.88, 0.97)	0.002	0.04
Hospital days	0.94 (0.87, 1.01)	0.08	0.15
Fluid-related hospitalizations	0.87 (0.79, 0.97)	0.01	0.06
Multivariable models			
All-cause hospitalizations	0.92 (0.88, 0.97)	0.002	0.03
Hospital days	0.93 (0.86, 1.00)	0.05	0.11
Fluid-related hospitalizations	0.86 (0.78, 0.96)	0.005	0.03

[1]P values were from the negative binomial model. [2] P values were from the negative binomial model, adjusting for the clinic clustering effect.

Supplemental Table S3A: Comparison of monthly average clinical measurements between HI-RBVM and NO-RBVM groups among all patients

Variables	Group	Month 1	Month 2	Month 3	Month 4	Month 5	Month 6	Month 6-1, p value[1]	P-value[2]	P-value[3]
Number of patients	HI-RBV	11826	10026	9320	8798	8329	7843	7840		
	NO-RBV	13108	10397	9658	9002	8430	7892	7887		
Treatment time (min, SD)	HI-RBVM	224.6 (24.7)	225.3 (24.6)	225.3 (24.8)	225.4 (24.9)	225.3 (24.9)	225.3 (24.9)	-0.5, p<0.001	<0.001	0.54
	NO-RBVM	223.8 (24.3)	224.9 (24.3)	224.8 (24.4)	224.6 (24.6)	224.4 (24.6)	224.4 (24.7)	-0.61, p<0.001	<0.001	
Pre-HD weight (kg, SD)	HI-RBVM	85.2 (23.9)	84.8 (23.8)	84.8 (23.8)	85 (23.8)	85 (23.7)	84.8 (23.6)	-0.5, p<0.001	<0.001	0.25
	NO-RBVM	86.2 (24.3)	85.5 (24.2)	85.5 (24.2)	85.6 (24.5)	85.5 (24.4)	85.5 (24.4)	-0.54, p<0.001	<0.001	
Post-HD weight (kg, SD)	HI-RBVM	83 (23.4)	82.6 (23.3)	82.5 (23.3)	82.6 (23.3)	82.6 (23.2)	82.5 (23.1)	-0.53, p<0.001	<0.001	0.15
	NO-RBVM	84.1 (23.9)	83.3 (23.8)	83.3 (23.7)	83.3 (24.0)	83.3 (23.9)	83.3 (23.9)	-0.57, p<0.001	<0.001	
IDWG (kg, SD)	HI-RBVM	2.14 (1.11)	2.27 (1.09)	2.26 (1.07)	2.3 (1.09)	2.37 (1.13)	2.26 (1.06)	-0.004, p=0.68	<0.001	0.57
	NO-RBVM	2.07 (1.16)	2.19 (1.11)	2.19 (1.14)	2.24 (1.19)	2.29 (1.2)	2.19 (1.11)	-0.02, p=0.07	<0.001	
EDW (kg, SD)	HI-RBVM	82.7 (23.5)	82.1 (23.2)	82 (23.1)	82.1 (23.1)	82.1 (23)	81.9 (22.9)	-0.72, p<0.001	<0.001	0.07
	NO-RBVM	83.8 (23.7)	82.8 (23.6)	82.8 (23.6)	82.8 (23.8)	82.7 (23.7)	82.8 (23.7)	-0.71, p<0.001	<0.001	
Ultrafiltration Volume (L, SD)	HI-RBVM	2.16 (1.02)	2.27 (1.01)	2.29 (1.01)	2.33 (1)	2.35 (1.05)	2.31 (1.01)	0.03, p<0.001	<0.001	0.36
	NO-RBVM	2.11 (1.05)	2.2 (1.07)	2.22 (1.06)	2.26 (1.02)	2.26 (1.40)	2.25 (1.01)	0.03, p<0.001	<0.001	
Ultrafiltration Rate (mL / kg / h, SD)	HI-RBVM	7.19 (3.25)	7.56 (3.17)	7.63 (3.11)	7.76 (3.16)	7.14 (3.06)	6.99 (3.20)	0.18, p<0.001	<0.001	0.15
	NO-RBVM	6.94 (3.31)	7.26 (3.34)	7.33 (3.38)	7.50 (3.15)	7.46 (6.45)	7.45 (3.10)	0.15, p<0.001	<0.001	
Pre-HD SBP (mmHg, SD)	HI-RBVM	145.2 (20.9)	145.8 (20.8)	146.6 (20.7)	147 (20.8)	147.7 (20.7)	147.6 (20.6)	1.64, p<0.001	<0.001	0.16

	NO-RBVM	145.5 (20.8)	146.0 (20.4)	146.7 (20.1)	147.6 (20.3)	148.1 (20.3)	148.2 (20.2)	1.75, p<0.001	<0.001	
Post-HD SBP (mmHg, SD)	HI-RBVM	138.6 (19.1)	138 (18.7)	137.7 (18.6)	136.7 (18.4)	136.8 (18.4)	136.5 (18.4)	-1.6, p<0.001	<0.001	0.07
	NO-RBVM	139.3 (19.1)	138.6 (18.5)	138 (18.2)	137.4 (18.1)	137.7 (18.1)	137.4 (18.0)	-1.56, p<0.001	<0.001	
On HTN medication, %	HI-RBVM	76.7	78.3	78	77.6	77.1	76.9	-1.88, p<0.001	<0.001	0.04
	NO-RBVM	76.3	79.7	79.6	79.1	78.6	78.3	-1.83, p<0.001	<0.001	
On ≥ 3 types of HTN medications, %	HI-RBVM	31.8	31.1	30.3	29.8	29	28.5	-3.55, p<0.001	<0.001	0.02
	NO-RBVM	31.6	32.2	31.6	31.1	30.8	30.4	-2.6, p<0.001	<0.001	

[1]P values were calculated using paired t-test to assess the significance of the differences between month 6 and month 1; [2]P values were calculated using the repeated-measures models to test the significance of monthly changes in each group; [3]P values were calculated from the repeated-measures models with interaction term to test whether the monthly changes were significantly different between HI-RBVM and NO-RBVM groups.

CHF, congestive heart failure; EDW, estimated dry weight; HD, hemodialysis; HI-RBV, high utilization of relative blood volume monitoring; HTN, hypertension; IDWG, interdialytic weight gain; NO-RBV, not using relative blood volume monitoring; SBP, systolic blood pressure; UFV, ultrafiltration volume.

Supplemental Table S3B: Comparison of monthly average clinical measurements between HI-RBVM and NO-RBVM groups among patients followed up for 6 months

**Variables**	**Group**	**Month 1**	**Month 2**	**Month 3**	**Month 4**	**Month 5**	**Month 6**	**Month 6-1, p value[1]**	**P-value[2]**	**P-value[3]**
Number of patients	HI-RBVM	7580	7546	7543	7541	7549	7583	7580		
	NO-RBVM	7608	7574	7569	7579	7580	7613	7608		
Treatment time (min, SD)	HI-RBVM	225.7 (24.6)	225.6 (24.7)	225.5 (24.8)	225.4 (24.9)	225.3 (24.9)	225.2 (24.9)	-0.50, p<0.001	<0.001	0.11
	NO-RBVM	225.1 (24.6)	225.1 (24.6)	224.9 (24.6)	224.7 (24.6)	224.6 (24.6)	224.5 (24.6)	-0.63, p<0.001	<0.001	
Pre-HD weight (kg, SD)	HI-RBVM	85.3 (23.8)	85.2 (23.8)	85.1 (23.7)	85.1 (23.6)	85.0 (23.6)	84.8 (23.6)	-0.48, p<0.001	<0.001	0.62
	NO-RBVM	86.2 (24.6)	86.1 (24.6)	86.0 (24.5)	86.0 (24.5)	85.9 (24.5)	85.7 (24.4)	-0.51, p<0.001	<0.001	
Post-HD weight (kg, SD)	HI-RBVM	83.0 (23.3)	82.9 (23.3)	82.8 (23.2)	82.7 (23.2)	82.6 (23.1)	82.5 (23.1)	-0.51, p<0.001	<0.001	1.00
	NO-RBVM	84.0 (24.1)	83.8 (24.1)	83.7 (24.0)	83.7 (24.0)	83.6 (24.0)	83.4 (23.9)	-0.54, p<0.001	<0.001	
IDWG (kg, SD)	HI-RBVM	2.27 (1.04)	2.34 (1.05)	2.30 (1.02)	2.34 (1.03)	2.39 (1.11)	2.27 (1.03)	-0.005, p=0.60	<0.001	0.55
	NO-RBVM	2.22 (1.04)	2.28 (1.04)	2.23 (1.09)	2.27 (1.05)	2.33 (1.06)	2.21 (1.09)	-0.01, p=0.18	<0.001	
EDW (kg, SD)	HI-RBVM	82.6 (23.2)	82.4 (23.1)	82.3 (23.1)	82.2 (23.0)	82.1 (23.0)	82.0 (22.9)	-0.68, p<0.001	<0.001	0.06
	NO-RBVM	83.6 (24.0)	83.4 (23.9)	83.3 (23.9)	83.2 (23.9)	83.1 (23.9)	82.9 (23.8)	-0.68, p<0.001	<0.001	
Ultrafiltration Volume (L, SD)	HI-RBVM	2.28 (1.00)	2.33 (0.99)	2.33 (1.00)	2.35 (0.99)	2.37 (1.04)	2.31 (1.00)	0.03, p<0.001	<0.001	0.13
	NO-RBVM	2.23 (1.01)	2.27 (0.99)	2.26 (1.04)	2.29 (1.01)	2.30 (1.01)	2.26 (1.00)	0.04, p<0.001	<0.001	
Ultrafiltration Rate (mL / kg / h, SD)	HI-RBVM	7.54 (3.11)	7.73 (3.04)	7.74 (3.04)	7.84 (3.12)	7.89 (3.24)	6.99 (3.20)	0.17, p<0.001	<0.001	0.08
	NO-RBVM	7.31 (3.13)	7.45 (3.02)	7.43 (3.32)	7.58 (3.08)	7.62 (3.12)	7.49 (3.07)	0.17, p<0.001	<0.001	
Pre-HD SBP (mmHg, SD)	HI-RBVM	146.0 (20.1)	146.5 (20.3)	147.1 (20.4)	147.4 (20.5)	147.9 (20.5)	147.7 (20.5)	1.68, p<0.001	<0.001	0.87
	NO-RBVM	146.5 (19.5)	146.9 (19.7)	147.2 (19.6)	148.1 (19.8)	148.5 (19.8)	148.3 (20.0)	1.87, p<0.001	<0.001	

Post-HD SBP (mmHg, SD)	HI-RBVM	138.0 (18.3)	137.8 (18.4)	137.6 (18.4)	136.7 (18.2)	136.8 (18.3)	136.5 (18.2)	-1.53, p<0.001	<0.001	0.17
	NO-RBVM	138.8 (18.1)	138.5 (18.1)	138.1 (17.9)	137.5 (17.8)	137.7 (17.8)	137.4 (17.8)	-1.48, p<0.001	<0.001	
On HTN medication, %	HI-RBVM	78.7	78.5	78.1	77.6	77.1	76.8	-1.92, p<0.001	<0.001	0.91
	NO-RBVM	80.2	80.0	79.6	79.1	78.7	78.4	-1.76, p<0.001	<0.001	
On ≥ 3 types of HTN medications, %	HI-RBVM	31.8	30.9	30.1	29.5	28.8	28.3	-3.5, p<0.001	<0.001	0.25
	NO-RBVM	33.0	32.3	31.5	31.2	30.8	30.4	-2.56, p<0.001	<0.001	

[1]P values were calculated using paired t-test to assess the significance of the differences between month 6 and month 1; [2]P values were calculated using the repeated-measures models to test the significance of monthly changes in each group; [3]P values were calculated from the repeated-measures models with interaction term to test whether the monthly changes were significantly different between HI-RBVM and NO-RBVM groups.

CHF, congestive heart failure; EDW, estimated dry weight; HD, hemodialysis; HI-RBV, high utilization of relative blood volume monitoring; HTN, hypertension; IDWG, interdialytic weight gain; NO-RBV, not using relative blood volume monitoring; SBP, systolic blood pressure; UFV, ultrafiltration volume.

Supplemental Table S4: Number of EDW changes over time in HI-RBVM and NO-RBVM groups among patients followed-up for 6 months.

**Study cohort**	**Group**	**Number of EDW changes, mean (SD)**	**P-value**
Overall (*n*=15,196)	HI-RBVM	6.15 (4.39)	<0.001
	NO-RBVM	5.29 (4.04)	
By hypertension in the first month			
No hypertension (*n*=5,856)	HI-RBVM	5.91 (4.33)	<0.001
	NO-RBVM	5.00 (3.86)	
Hypertension (*n*=9,332)	HI-RBVM	6.31 (4.43)	<0.001
	NO-RBVM	5.47 (4.13)	

EDW, estimated dry weight; HI-RBV, high utilization of relative blood volume monitoring; NO-RBV, not using relative blood volume monitoring; SD, standard deviation.
